# Marginal lands and fungi – linking the type of soil contamination with fungal community composition

**DOI:** 10.1111/1462-2920.16007

**Published:** 2022-05-30

**Authors:** Alicja Okrasińska, Przemyslaw Decewicz, Maria Majchrowska, Lukasz Dziewit, Anna Muszewska, Somayeh Dolatabadi, Łukasz Kruszewski, Zuzanna Błocka, Julia Pawłowska

**Affiliations:** ^1^ Institute of Evolutionary Biology, Centre of Biological and Chemical Research Centre, Faculty of Biology University of Warsaw Warsaw Poland; ^2^ Department of Environmental Microbiology and Biotechnology, Institute of Microbiology, Faculty of Biology University of Warsaw Warsaw Poland; ^3^ Institute of Biochemistry and Biophysics Polish Academy of Sciences Warsaw Poland; ^4^ Department of Biology Hakim Sabzevari University Sabzevar Iran; ^5^ Institute of Geological Sciences Polish Academy of Sciences Warsaw Poland

## Abstract

Fungi can be found in almost all ecosystems. Some of them can even survive in harsh, anthropogenically transformed environments, such as post‐industrial soils. In order to verify how the soil fungal diversity may be changed by pollution, two soil samples from each of the 28 post‐industrial sites were collected. Each soil sample was characterized in terms of concentration of heavy metals and petroleum derivatives. To identify soil fungal communities, fungal internal transcribed spacer 2 (ITS2) amplicon was sequenced for each sample using Illumina MiSeq platform. There were significant differences in the community structure and taxonomic diversity among the analysed samples. The highest taxon richness and evenness were observed in the non‐polluted sites, and lower numbers of taxa were identified in multi‐polluted soils. The presence of monocyclic aromatic hydrocarbons, gasoline and mineral oil was determined as the factors driving the differences in the mycobiome. Furthermore, in the culture‐based selection experiment, two main groups of fungi growing on polluted media were identified – generalists able to live in the presence of pollution, and specialists adapted to the usage of BTEX as a sole source of energy. Our selection experiment proved that it is long‐term soil contamination that shapes the community, rather than temporary addition of pollutant.

## Introduction

Fungi are a diverse and abundant group of eukaryotic organisms and their representatives are found in almost all ecosystems (Bar‐On *et al*., 2018), including deep oceans (Nagano and Nagahama, 2012), deserts (Sun *et al*., 2019) and the permafrost of Antarctica (de Menezes *et al*., 2020). They are abundant not only in various pristine habitats but they can also thrive in anthropogenically shaped habitats, like urban soils (Baruch *et al*., 2020), post‐industrial sites (Thion *et al*., 2012) and even jet fuel (Itah *et al*., 2009). With parasitic and mutualistic symbionts, as well as saprotrophs among them, they play many important roles in ecosystems (Robson, 2017; Větrovský *et al*., 2019). Due to their abilities to produce various extracellular enzymes, fungi are responsible for decomposition of organic matter in soil and thus play a crucial role in nutrient cycling and pedogenesis in all types of soils (Bardgett and van der Putten, 2014).

Recent development of high‐throughput sequencing has facilitated the analysis of soil fungal communities (Frąc *et al*., 2018; Landinez‐Torres *et al*., 2019; Delgado *et al*., 2021) and enabled their high throughput, global comparisons (Tedersoo *et al*., 2014; Egidi *et al*., 2019; Větrovský *et al*., 2019; Baldrian *et al*., 2021) leading to a better understanding of the functioning of soil fungal communities. The analysis of several amplicon‐based datasets in global scale showed that Ascomycota encompassed the largest proportion of sequences in the soil (55%–70%, depending on the dataset). Functionally, almost half of detected fungi were assigned as saprotrophs. However, significant differences in taxonomical and functional composition were observed between different sites (Tedersoo *et al*., 2014; Panelli *et al*., 2017; Větrovský *et al*., 2019; Nicola *et al*., 2021). Community composition was also proven to change over time (Dresch *et al*., 2019; Liu and Howell, 2021), as it is shaped by several biotic and abiotic factors that are frequently interconnected (Tokeshi, 1990). Soil fungal communities were shown to strongly depend on the vegetation type (Thion *et al*., 2012; Bourceret *et al*., 2016; Hui *et al*., 2017), acidity, organic matter content and climatic conditions (Tedersoo *et al*., 2014; Větrovský *et al*., 2019; Shen *et al*., 2020; Bahram *et al*., 2021).

Organic and inorganic pollutants may also play a relevant role in shaping soil fungal communities (Cachada *et al*., 2018). Their presence in the environment may be a consequence of natural processes (e.g. volcanic activity, erosion, or forest fires), but most of them have evolved due to anthropogenic activities (Dhaliwal *et al*., 2020). This includes both heavy metals and petroleum‐derived contaminants, such as polycyclic aromatic hydrocarbons (PAHs) and monocyclic aromatic hydrocarbons (BTEX – benzene, toluene, ethylene and xylene isomers), which are considered the most detrimental environmental pollutants (Bourceret *et al*., 2016; Gałązka *et al*., 2020). Despite that, some fungi are capable of growth in the presence of all these pollutants, some may even be able to use them as a carbon source (Prenafeta‐Boldú *et al*., 2002). Fungi that secrete extracellular enzymes primarily used for cellulose and lignin decomposition (e.g. cytochrome P450, lignin peroxidase, manganese peroxidase and laccase) can be exploited for degradation of various organic pollutants, including PAHs (Baldrian, 2003; Harms *et al*., 2011). Although these enzymes are most effectively produced by white‐rot fungi (e.g. Fulekar *et al*., 2013), they are also synthesized by representatives of other ubiquitous fungal groups, like Mucoromycota (Lisowska *et al*., 2006) and Ascomycota (Aranda, 2016). Some fungi may also transform heavy metal ions to their less toxic forms, while others produce siderophores that form complexes with heavy metals and can also play a significant role in bioremediation of other pollutants.

The long‐term experiments performed on multi‐polluted, post‐industrial sites demonstrate that fungal communities in this type of soil are dominated by Ascomycota (Thion *et al*., 2012; Bourceret *et al*., 2016). Recent studies of Gałązka *et al*. (2020) and Galitskaya *et al*. (2021) reported also Basidiomycota (e.g. *Hypholoma*, *Coprinellus*) and Mortierellomycota representatives as characteristic for industrially polluted soils. Slow‐growing ascomycetous microfungi from the *Knufia*, *Exophiala*, *Cladophialophora* and *Phialophora* genera, often labelled as ‘black yeasts’, also thrive in petroleum‐polluted areas (Dolatabadi *et al*., 2019; Gałązka *et al*., 2020). There is also evidence that overall fungal diversity increases with time after the withdrawal of the mining activity (Thion *et al*., 2012; Bourceret *et al*., 2016). The emerging fungal community is strongly linked with the succession of plants and fungi developing either from indigenous resting spores or from surrounding areas (Malloch and Blackwell, 1992; Thion *et al*., 2012).

To investigate the influence of the anthropogenic pollutants on soil mycobiota, we performed a comparison of soil fungal diversity between multi‐polluted industrial sites and non‐polluted regions with special emphasis on the adaptation and selection process. We hypothesized that while a large number of evenly represented taxa would be characteristic for the soil from the control sites, certain species of well‐adapted specialists would dominate in polluted areas. Finally, we aimed to replicate this contamination‐driven selection process under the laboratory conditions, in order to empirically demonstrate to what extent, the diversity may be shaped by soil pollution.

## Results

### The comparison of edaphic characters between sampling sites shows significant differences

**Fig. 1:**
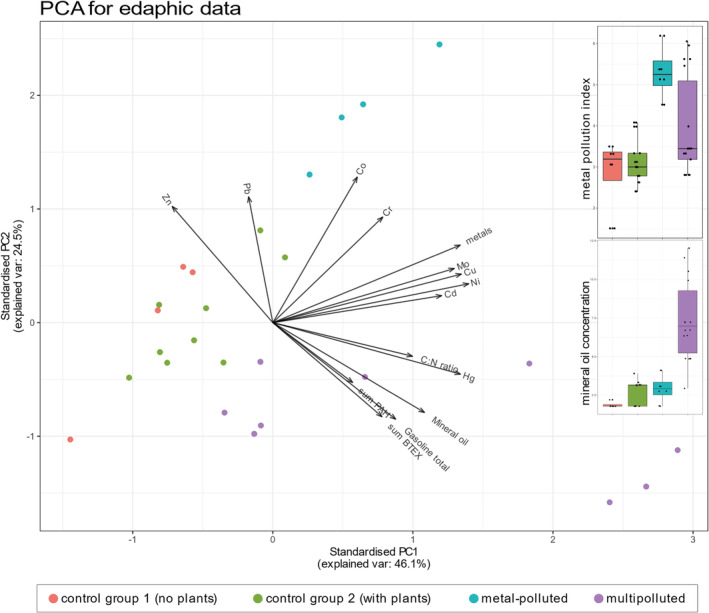
PCA biplot based on the numeric part of edaphic characteristics of the samples. All values were logarithmically transformed prior to plotting. Boxplots in the corner represent differences in concentrations of selected contaminants between groups.

Measurements of edaphic characteristics for 52 samples from 25 locations in Poland and three in Iran (Supplementary Table 1, Supplementary Fig. 1) were utilized to cluster the samples. PCA was used to visually represent the grouping (Fig. 1). Two first principal components explained 70.6% of variance. The following groups were delimited: (i) control sites without plants, (ii) control sites with plants, (iii) metal‐polluted areas, characterized by increased content of Zn, Pb, Co and Cr ions and (iv) multi‐polluted sites, characterized by increased presence of mineral oils, gasoline, BTEX, PAHs and mercury. This grouping of soil samples based on the numeric part of edaphic data was justified by ANOVA (anosim) test with 999 permutations (*R* ^2^ = 0.4784, *p* = 0.001) which confirmed that there are differences between these groups (Fig. 1).

### The fungal community structure differs between polluted and control sites

From the Illumina sequencing, 10 909 541 paired‐end reads were obtained (Supplementary Table 2, rarefaction curves can be found in Supplementary Fig. 2). After quality evaluation, which included denoising, length and quality trimming, and chimeric sequence exclusion, 5 506 508 sequences remained and were assigned into 9691 amplicon sequence variants (ASVs, Supplementary Table 2). After identifying and removing erroneous and redundant ASVs using LULU, 5005 ASVs remained.

**Fig. 2:**
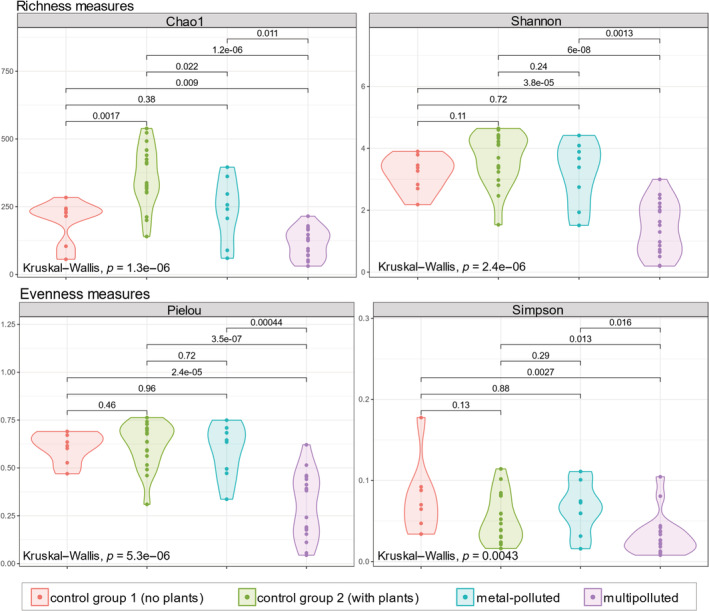
Alpha diversity plots for each of the sample groups. Top two plots show richness measures (Chao1 index on the left and Shannon index on the right). Bottom two plots represent evenness measures (Pielou index on the left and Simpson index on the right). Significance of the differences between groups in each measure (Kruskal–Wallis test) is shown above the line connecting groups. In the bottom left corner of every plot, the significance of the global Kruskal–Wallis test is denoted.

Differences in taxon richness (Chao1 and Shannon measures) between delimited groups (Fig. 2) were predominantly statistically significant (*p* < 0.005) as measured by Kruskal–Wallis test. However, the difference in fungal species richness and evenness between control sites without vegetation and metal‐polluted ones was not statistically significant. The highest richness was observed in control sites covered with vegetation, while the samples from multi‐polluted sites were the least taxon rich. At the same time, these samples were characterized by significant dominance of certain taxa (Pielou and Simpson evenness measures), while ASVs distribution in other groups was more even (Fig. 2).

**Fig. 3:**
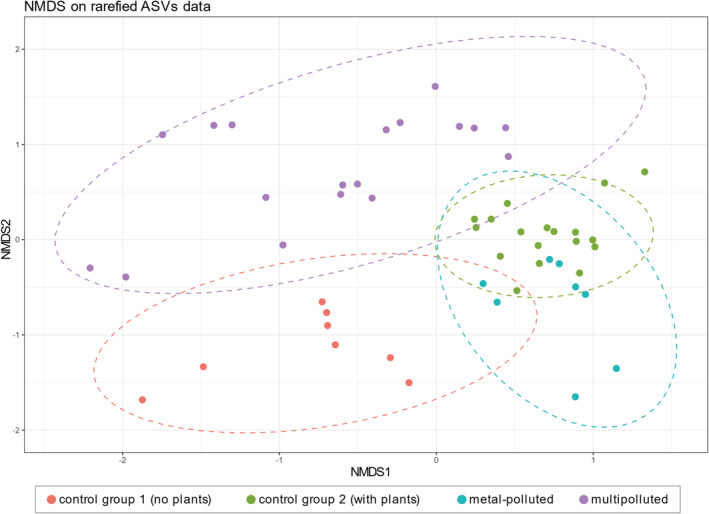
NMDS plot showing variation of ASV communities within groups of soil samples. Prior to plotting, ASV communities were rarefied to even depth.

The differences between delimited groups are also pronounced in taxonomic composition of analysed fungal communities. The phylum represented by the greatest number of ASVs (1693) and with the highest relative abundance (66.76%) was Ascomycota. Relative abundances of ascomycetous sequences in the soil samples range from 15.29% to 99.86% (Supplementary Table 3). The grouping of sampling sites by non‐metric multidimensional scaling (NMDS) of ASVs composition reflected the one based on edaphic parameters of soils (Fig. 3). The significance of differences in taxonomic composition between groups was confirmed by a one‐way ANOVA test (adonis), *F*(3,48) = 3.0976, *p* = 0.001.

The correlation between soil parameters and fungal species composition was measured using Mantel test based on Euclidean distance matrices. Total gasoline, total BTEX, mineral oil, and mercury concentrations, as well as carbon to nitrogen ratio, were the factors that correlated (Spearman correlation coefficient; *p* = 0.001) with fungal ASVs composition. Therefore, these factors may be treated as the ones shaping soil fungal communities, rather than metallic contaminants on their own.

**Fig. 4:**
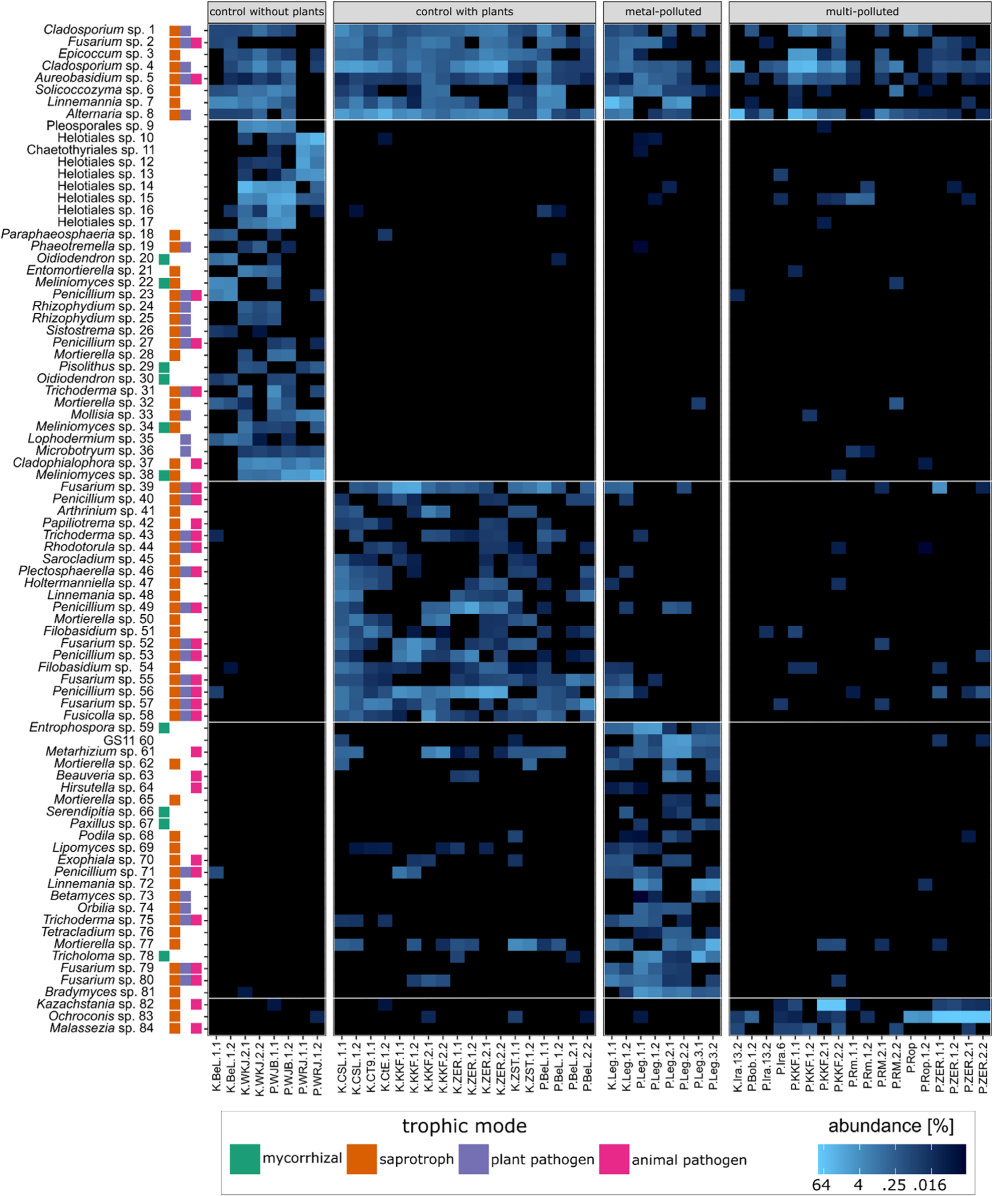
Heatmap representing relative abundances of the generalists and indicator taxa (defined in the Methods section) in each sample. Horizontal white lines divide groups of generalists and indicator taxa for each group. On the left side of the plot, the taxonomical identification and trophic modes are given.

The indicator ASVs for each of the delimited groups were also identified (Fig. 4) as described in the Methods section. Generalists were recognized if they were present (with abundance ≥5) in more than half of the samples. These taxa belonged to the following genera: *Alternaria*, *Aureobasidium*, *Cladosporium*, *Epicoccum*, *Fusarium*, *Linnemannia* and *Solicoccozyma*. Furthermore, the highest number of characteristic ASVs was detected for control sites without vegetation (30, numbers 9–38 in Fig. 4), while only few indicator taxa were detected in multi‐polluted sites (3, numbers 82–84 in Fig. 4). The ASVs shown to be characteristic for multi‐polluted sites represented the *Malassezia*, *Ochroconis* and *Kazachstania* genera, while ones typical for metal‐polluted (numbers 59–81 in Fig. 4) sites were representatives of the *Bradymyces*, *Entrophospora*, *Mortierella*, *Trichoderma*, *Serendipitia*, *Hirsutella*, *Metarhizium* and *Beauveria* genera. Although the majority of identified indicator taxa were Ascomycota representatives, their percentage rate ranged from 52% for metal‐polluted sites to 100% for multi‐polluted ones. The control soils without vegetation were characterized by the abundance of indicator taxa from the *Helotiales* order. The only *Glomeromycotina* indicator ASV (*Entrophospora* sp. 59 in Fig. 4) was typical for metal‐polluted soils, probably due to the herbaceous vegetation cover of these locations. Additionally, representatives of the GS11 clade (no. 60 in Fig. 4) of unidentified fungi from *Rozellomycota* (as defined by Tedersoo *et al*., 2017) were also selected as characteristic for metal‐polluted sites. *Mortierellomycotina* representatives were typical for control (e.g. *Entomortierella* sp. 21 and *Mortierella* sp. 28 and 32 in Fig. 4) and metal‐polluted sites (e.g. *Mortierella* sp. 62, 65 and 77, *Podila* sp. 68, and *Linnemannia* sp. 72 in Fig. 4).

### Fungal communities' short‐term adaptations to specific pollutants

In the culture‐based experiment, in which three mixtures of soil samples (detailed description can be found in the Experimental procedures section) were cultured on control (no pollutant added) and contaminated agar media, extensive fungal growth was observed on all MEA‐based media, while it was very limited on plates with BTEX as sole carbon source. No growth was observed on plates with BTEX as a sole carbon source inoculated with soil that originated from multi‐polluted sites. From the Illumina sequencing of 11 remaining sample sets, 4 862 646 paired‐end reads were obtained. After the processing, same as described for the main experiment, 2 891 220 assembled reads were analysed and assigned into 290 ASVs (Supplementary Table 2). After LULU processing (analogous to the processing in the main experiment), 84 ASVs remained.

**Fig. 5:**
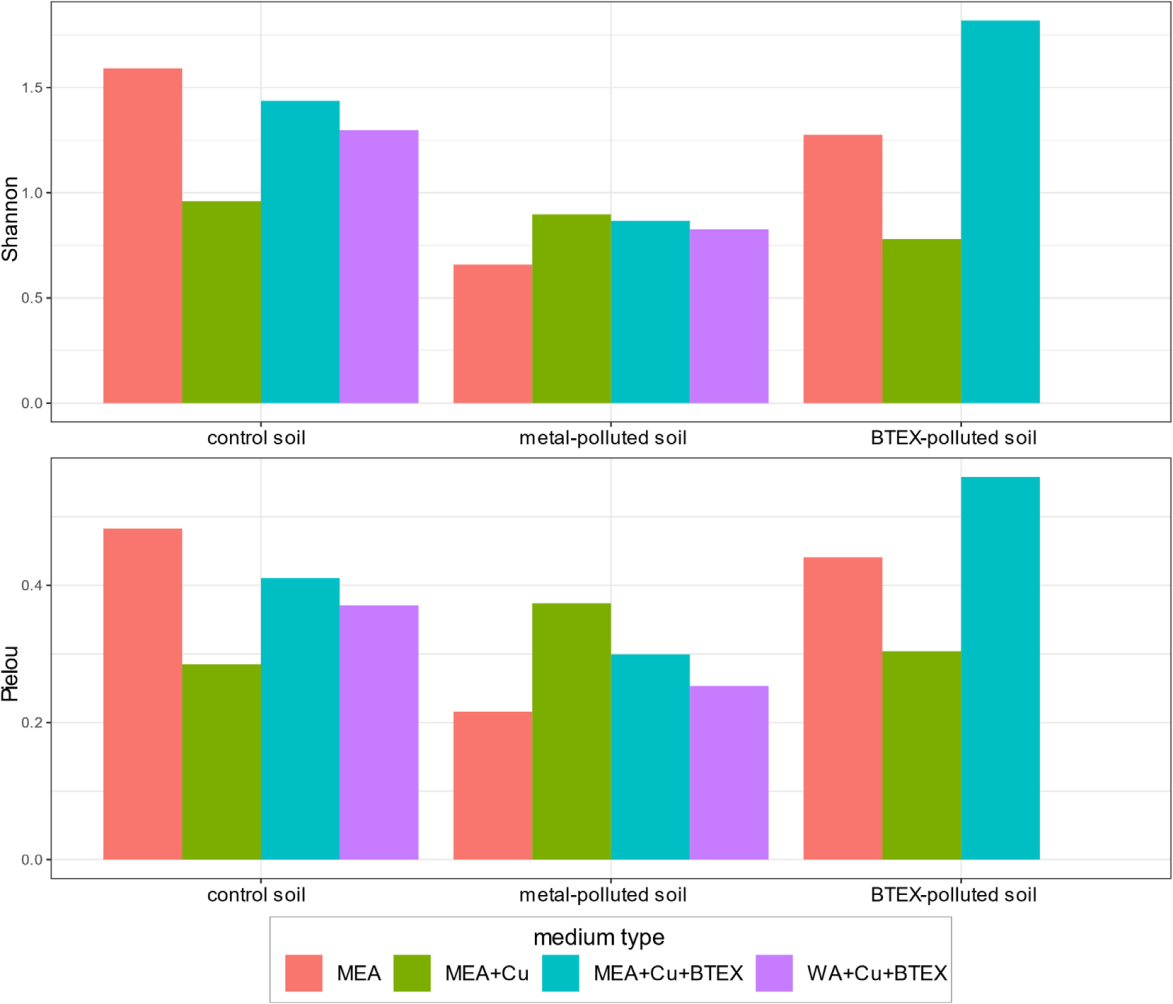
Barplots representing ASV richness (top, Shannon index) and evenness (bottom, Pielou index) in the samples. Each group of bars represents one type of soil sample inoculated on four different media types [red: pure MEA, green: MEA with copper salts added, blue: MEA with copper salts and aromatic hydrocarbons added, purple: WA with copper salts and aromatic hydrocarbons added]. No product was obtained for BTEX‐polluted soil samples inoculated on water agar with copper salts and BTEX.

**Fig. 6:**
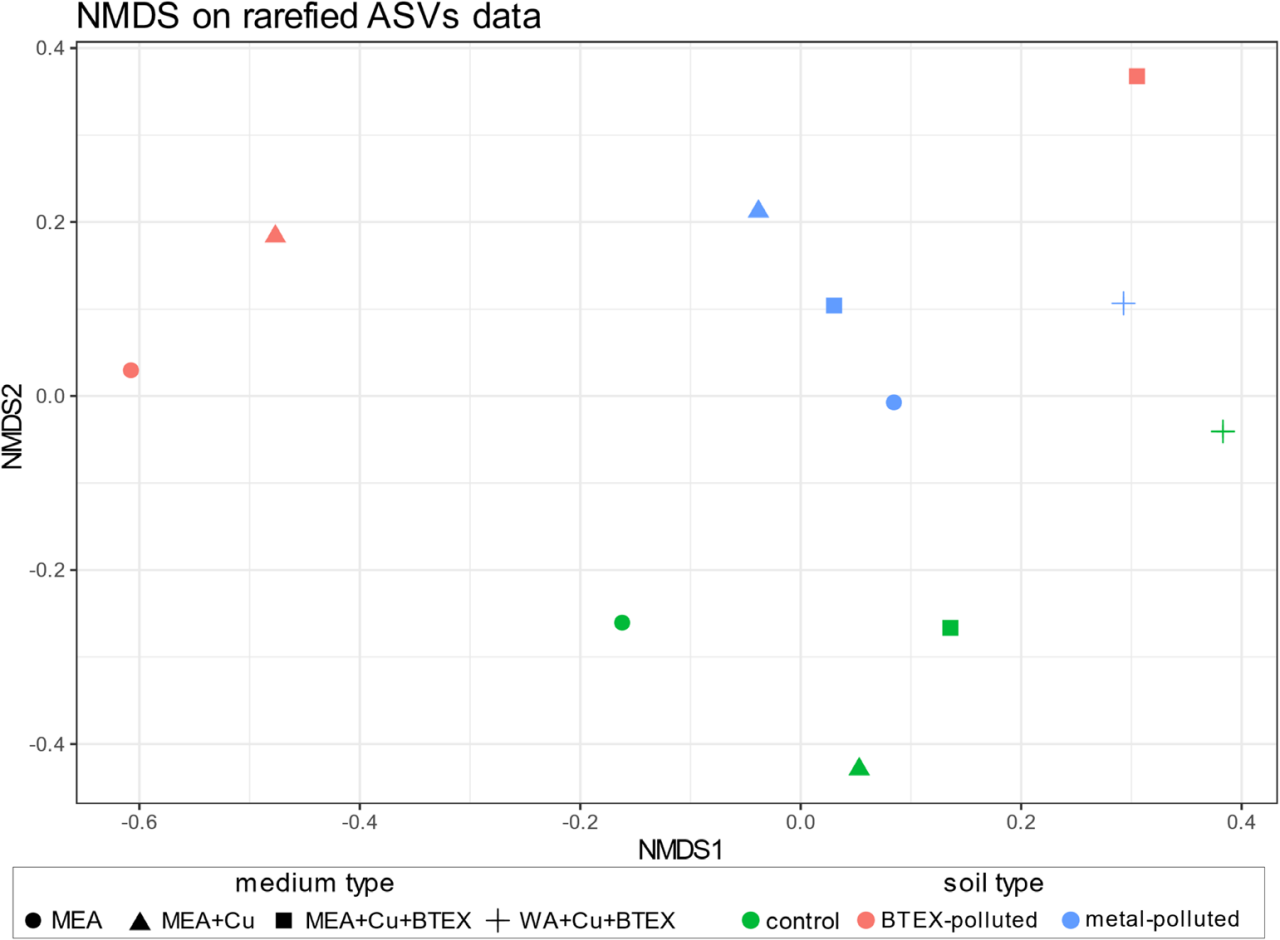
NMDS plot showing variation of ASV communities from polluted and non‐polluted soil samples after inoculating them on different media. Colours of the points represent the type of contamination of the soil from which the samples were taken (green: non‐contaminated sites, red: aromatic hydrocarbons‐contaminated sites, blue: metal‐contaminated sites). Shapes represent the type of agar medium on which samples were cultured [circle: pure MEA, triangle: MEA with copper added, square: MEA with copper and aromatic hydrocarbons added, cross: WA with copper and aromatic hydrocarbons added]. Prior to plotting, ASV communities were rarefied to even depth.

**Fig. 7:**
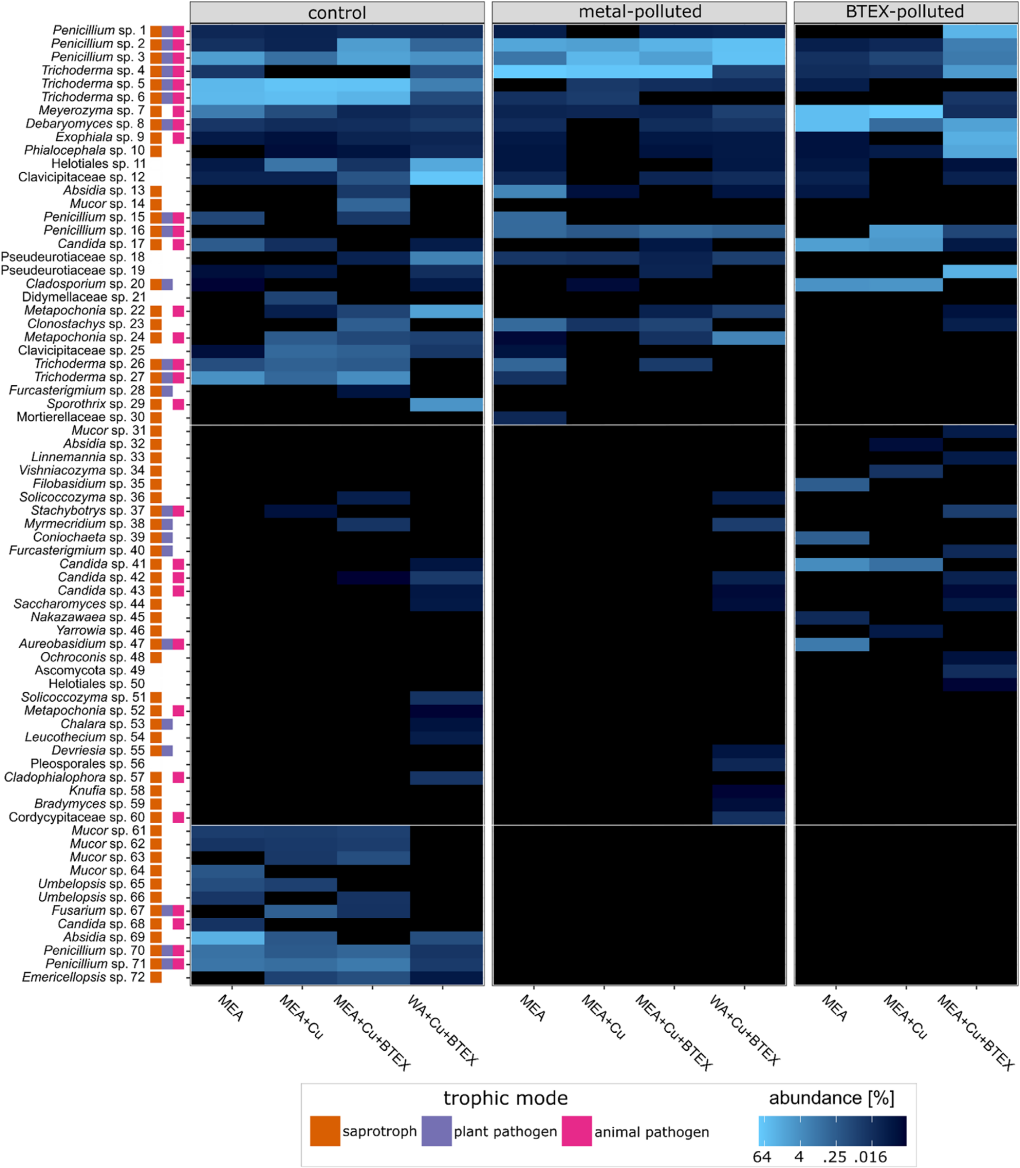
Heatmap representing relative abundances of the fungal ASVs derived from culturing differently polluted soils samples on four different media. Horizontal white lines divide groups (from the top) of generalists, BTEX‐tolerant fungi and fungi present only in non‐polluted sites. On the left side of the plot, the taxonomical identification and trophic mode are given.

The differences in taxon richness (Chao1 and Shannon measures) and evenness (Pielou and Simpson evenness measures) were observed between different growth media (Fig. 5). However, due to small sample sizes, it was impossible to measure the statistical significance of these differences. On culture media without addition of pollutants, the highest taxon richness and evenness were observed for samples from control sites. Moreover, high alpha diversity parameters were observed in samples from multi‐polluted sites cultured on a rich medium supplemented with copper salts and BTEX mixture. This phenomenon can be explained by the fact that all samples from multi‐polluted sites contained the same propagule set, meaning they all had potential to develop the same fungal community. However, in some cases, once the BTEX mixture was added to culture media, generalists (like *Cladosporium* sp. 20 in Fig. 7) were not able to develop, while the otherwise slow‐growing extremotolerant organisms (e.g. *Ochroconis* sp. 48 in Fig. 7) had a chance to expand. This adaptation pattern can also be observed when analysing the Pielou evenness measures. Taxonomic evenness was highest when added contaminant corresponded with the type of pollutant in the original soil sample. Similarly, the taxonomic composition of soil fungal communities depended on soil origin more than on the pollutant addition (Fig. 6, confirmed by one‐way ANOVA *p* = 0.001 and *p* = 0.968 respectively). Another interesting trend which can be observed in Fig. 5 is that in control and BTEX‐polluted sites, addition of copper salts to the medium resulted in lower richness and evenness of fungal community, whereas further addition of BTEX mixture increased both these measures. It shows that BTEX serves rather as an energy source than growth inhibitor for some fungi (e.g. *Absidia* sp. 13 or *Linnemannia* sp. 33 in Fig. 7). Although the group of generalists able to develop on plates with BTEX as a sole carbon source was similar for all types of soils (including *Exophiala* sp. 9, *Penicillium* sp. 1–3, *Trichoderma* sp. 4, 6 in Fig. 7), some unique taxa were also detected in each soil type, including, e.g. *Emericellopsis* sp. 72, *Absidia* sp. 69 and *Penicillium* sp. 70 for control soils, and *Bradymyces* sp. 59 and an unidentified *Cordycipitaceae* (60) representative for metal‐polluted soils (Fig. 7). These are in concordance with the results from the field diversity study because the same taxa were detected as indicator ASVs for metal‐polluted soils in both experiments.

Most of the analysed samples were dominated by generalists such as *Penicillium* spp., *Trichoderma* spp., *Phialocephala* sp., *Exophiala* sp. and yeasts belonging to the *Meyerozyma* and *Debaryomyces* genera. However, the differences in their prevalence were observed between samples, supporting the hypothesis on specific adaptations of fungal communities. For example, the control plates (MEA without the addition of pollutants) inoculated with soil from control sites were characterized by extensive presence of the *Trichoderma*, *Penicillium* and *Absidia* representatives, whereas the addition of copper salts and BTEX eliminated organisms such as *Mucor* sp. 64, *Candida* sp. 68, or *Metapochonia* sp. 22 and 24 while favouring *Phialocephala* sp. 10 and *Emericellopsis* sp. 72 (Fig. 7). On the other hand, the control plates inoculated with soil from metal‐polluted sites were characterized by the dominance of *Trichoderma* and *Penicillium* that were not affected by further addition of copper salts or BTEX (e.g. *Trichoderma* sp. 5, *Penicillium* sp. 2 and 3 in Fig. 7). Finally, on plates with BTEX as sole carbon source, samples from control sites represented the most even and species‐rich fungal communities. This community consisted, among else, of *Clavicipitaceae* (*Metapochonia* sp. 24 and 52, *Clavicipitaceae* sp. 12), *Ophiostomataceae* (*Sporothrix* sp. 29) and yeasts (e.g. *Candida* sp. 41–43 and *Solicoccozyma* sp. 51 in Fig. 7) *–* the taxa that are usually outgrown under optimal conditions but become dominant when the sole carbon source is BTEX.

## Discussion

### Fungal communities in polluted soils – selection and adaptation processes

The soil fungal communities from multi‐polluted sites were characterized by the lowest alpha diversity measures (taxon richness and evenness), proving that mercury and oil derivatives pollutants play an important role in shaping these communities. This finding is in line with some results of previous studies (e.g. Bell *et al*., 2014; Bourceret *et al*., 2016). Based on the soil fungal diversity analysis, the presence of the hydrocarbons has a higher impact on the richness and taxonomic composition of fungal community than the presence of heavy metals, as indicated by both quantitative and qualitative data. A similar strong influence of multi‐pollution on soil fungal communities has already been demonstrated by other authors (e.g. Thion *et al*., 2012; Bourceret *et al*., 2016).

Fungal communities, present in multi‐polluted sites, were characterized by the highest share of Ascomycota representatives (in 12 out of 19 samples from this group there were more than 90% of Ascomycota sequences, see: Supplementary Table 3). Similar pattern was observed in long‐term experiments performed on multi‐polluted, post‐industrial sites. In fact, Cébron *et al*. (2009), Thion *et al*. (2012) and Bourceret *et al*. (2016) observed that Basidiomycota representatives make up a smaller percentage of fungal community in this type of soil than in non‐polluted sites.

Owing to large sampling in our study, the statistically supported delimitation of fungal communities typical for each particular soil contamination type was possible, and we were able to identify indicator species associated with a given type of pollution. The ASVs shown to be characteristic for multi‐polluted sites represented *Malassezia*, *Ochroconis* and *Kazachstania* genera, while ones typical for metal‐polluted sites were representatives of *Bradymyces*, *Entrophospora*, *Mortierella*, *Trichoderma*, *Serendipitia*, *Hirsutella*, *Metarhizium* and *Beauveria* genera. All of these taxa were previously reported from contaminated soils. However, taxa often indicated in the literature as typical for oil‐contaminated soils, such as *Knufia*, *Exophiala*, *Cladophialophora* and *Phialophora* (Dolatabadi *et al*., 2019; Gałązka *et al*., 2020), while being present in the samples, were not selected as indicator taxa for multi‐polluted soil (see Supplementary Table 3 in comparison with Fig. 4). These taxa are slow‐growing extremotolerants that are able to develop in sites polluted with oil derivatives, but also occur in other habitats (Teixeira *et al*., 2017; Costa *et al*., 2020) where their presence is more difficult to notice due to the dominance of fast‐growing representatives of Ascomycota, such as *Penicillium* or *Trichoderma*. This phenomenon was confirmed in our culture‐based experiment (see Fig. 7).

Bourceret *et al*. (2016) hypothesized that while a short‐term impact of contamination tends to decrease microbial abundance, richness and diversity, the long‐term contamination leads to successive selection of unique and relatively diverse microbial communities adapted to particular types of pollutants. Our culture‐based experiment confirmed this hypothesis, as evenness was highest when added contaminant corresponded with the type of pollutant in the original soil sample.

### Metal‐specific fungal communities

Metal ions have been known to impact fungal biology, serving as micronutrients in small doses. In excessive amounts, however, some of them can be used as antifungal agents as they can disrupt fungal homeostasis. Some fungi have thus developed resistance mechanisms against high concentrations of these pollutants. These mechanisms include binding protein modifications, efflux pumps, overproduction of binding proteins, vacuole sequestration, as well as various detoxifying mechanisms (reviewed by Robinson *et al*., 2021). Although most of the toxicity and resistance studies are performed on yeasts, there is some research on the capabilities of filamentous fungi, mostly Ascomycota, to absorb and detoxify certain metals (lead and copper: Iskandar *et al*., 2011, zinc: Teng *et al*., 2018, cobalt: Cárdenas González *et al*., 2019). Most of these studies focus on well‐studied and easy to cultivate fungi, such as *Aspergillus* spp., *Penicillium* spp., *Fusarium* spp., or *Trichoderma* spp. In our study, one *Penicillium* ASV (no. 71 in Fig. 4), one *Trichoderma* ASV (no. 75 in Fig. 4) and two *Fusarium* ASVs (79 and 80 in Fig. 4) were identified as indicators for metal‐polluted sites.

The taxa identified as indicators of metal (zinc, lead, cobalt and chromium) polluted soils in our study include a few entomopathogenic fungi (i.e. *Metarhizium* sp. 61, *Beauveria* sp. 63 and *Hirsutella* sp. 64 in Fig. 4). Some of the entomopathogenic taxa, e.g. *Beauveria bassiana*, have been previously shown to effectively remove heavy metals from soil via sorption and accumulation processes (Gola *et al*., 2016). Another entomopathogenic fungus, *Metarhizium anisopliae*, was shown to use its host's chitin to produce chitosan (de Assis *et al*., 2010), which has been used as an adsorbent of heavy metal ions and organic compounds (Peniche‐Covas *et al*., 1992). Therefore, the extensive presence of entomopathogenic taxa in metal‐polluted areas can be explained by several specific adaptation mechanisms of this group of fungi.

Among the indicator taxa were also four exclusively mycorrhizal fungi, including an arbuscular mycorrhizal fungus – *Entrophospora* sp. 59 in Fig. 4 (Glomeromycota) – and three basidiomycetous cap fungi which form ectomycorrhizae with plants (namely, *Serendipitia* sp. 66, *Paxillus* sp. 67 and *Tricholoma* sp. 78 in Fig. 4). Arbuscular mycorrhizal fungi (AMF) were previously shown to aid plants when growing in heavy metal‐polluted soil by accumulating part of the pollution in their structures (review by Riaz *et al*., 2021). The AMF were shown to be able to survive extremely high concentrations of heavy metals (Sánchez‐Castro *et al*., 2017) and they are commonly used to increase metal phytoremediation potential of some plants (e.g. Chaturvedi *et al*., 2021). *Entrophospora* sp., the AMF detected as indicator taxa in our study, was also previously found in close proximity of a copper mine in Brazil (da Silva *et al*., 2005). On the other hand, there are very few studies on the benefits of having ectomycorrhizal partners for plants under heavy metal stress, and their presence in the samples can be probably explained by tree roots' presence in the sampling plots. However, a recent study has shown that pine seedlings grow more efficiently in heavy metal‐polluted soil when they form ectomycorrhiza (Hachani *et al*., 2020).

### Fungal communities of petroleum derivatives‐contaminated soils

Hydrocarbons have been present in nature for a long time (i.e. produced by plants; Giger and Blumer, 1974). However, introduction of petroleum‐based fuels made them more ubiquitous and therefore more problematic. Some bacteria, archaea and fungi have developed metabolic and physiological adaptations to survive in the presence of hydrocarbons, as well as to directly utilize them (Asperger and Kleber, 1991; Robertson *et al*., 2007; Daccò *et al*., 2020). Biological degradation of hydrocarbons can be partial, resulting in partially oxidized intermediates, or complete, when catabolic products are water and carbon dioxide (Abbasian *et al*., 2015). Aromatic hydrocarbons are usually metabolized through one of the following oxidation processes: (i) intracellular cytochrome P450 monooxygenases activity, (ii) excreted laccases' activity, or (iii) excreted lignin‐degrading peroxidases' activity, which all result in partially oxidized intermediates (Prenafeta‐Boldú *et al*., 2018). However, aliphatic hydrocarbons, which are the main component of modern fuels, can be degraded by fungi using the first process (Scheller *et al*., 1998).

Lignin peroxidases are mainly known from basidiomycetous fungi, but their presence in the cosmopolitan fungi, such as *Aspergillus*, *Penicillium* and *Fusarium* genera, was also confirmed (Rodríguez *et al*., 1996). ASVs representing these taxa were found not only in multi‐polluted soils contaminated with aliphatic hydrocarbons (Fig. 4, Supplementary Table 3) but also on medium with BTEX as the only carbon source (Fig. 7).

Laccases are more common in the fungal kingdom, as these enzymes also play a role in the formation of fungal melanin (Mayer and Staples, 2002). The representatives of *Dothideales* genera *Aureobasidium* and *Cladosporium* were shown to degrade PAHs by laccases secretion (Potin *et al*., 2004; Leelaruji *et al*., 2014), while the representatives of *Chaetothyriales* fungi, such as *Exophiala*, are often isolated from sites that are polluted with monoaromatic hydrocarbons, e.g. BTEX (Prenafeta‐Boldú *et al*., 2001a). In our experiments, several of these highly melanized taxa representatives were shown to be able to utilize aromatic pollutants for their own growth, as they grew on agar plates where BTEX mix was the sole carbon source (see Fig. 7, e.g. *Exophiala* sp. 9, *Cladophialophora* sp. 57, *Knufia* sp. 58 and *Bradymyces* sp. 59).

The intracellular cytochrome P450 monooxygenases, able to oxidize PAHs, were described in detail in *Phanerochaete chrysosporium* and *Cunninghamella* sp. (Juhasz and Naidu, 2000; Asha and Vidyavathi, 2009; Cerniglia and Sutherland, 2010; Syed *et al*., 2013). However, some other taxa detected in our study, namely, *Beauveria*, *Penicillium*, *Exophiala*, *Cladosporium* and *Cladophialophora*, were also previously reported to grow on toluene, ethylbenzene and styrene (Fedorak and Westlake, 1986; Prenafeta‐Boldú *et al*., 2018), degrading them by oxidizing alkyl groups by specific CYP monooxygenases (Weber *et al*., 1995; Cox *et al*., 1996; Prenafeta‐Boldú *et al*., 2001b; Prenafeta‐Boldú *et al*., 2002; Luykx *et al*., 2003). Interestingly, the same pathway is used by several bacteria able to metabolize n‐alkylbenzenes (Finette *et al*., 1984). Blasi *et al*. (2017) validated the existence of this metabolic pathway in a transcriptomic analysis of *Cladophialophora* sp., claiming that it was acquired from *Pseudomonas*‐related bacteria by horizontal gene transfer.

As fungi, bacteria and other soil microorganisms coexist in the same niche, they often form alliances. These interkingdom interactions can be physical, when fungal hyphae facilitate the movement of bacteria, acting as ‘highways’ (Warmink *et al*., 2011), but often are also metabolic. For example, fungi secrete extracellular enzymes which partially degrade various polymers (Boer *et al*., 2005). Products of these processes can be further utilized as an energy source by other microorganisms present in the soil. Microbiomes of the soil, of which fungi and bacteria are important components, are still poorly studied (Zegeye *et al*., 2019). Therefore, synergistic interactions between fungi and bacteria which can result in a complete biodegradation of hydrocarbons need to be further studied, as was already pointed out by Prenafeta‐Boldú *et al*. (2018). The fungal group well known for its common and intimate interactions with bacteria, even on endohyphal level, is Mucoromycota phylum (Bonfante and Desirò, 2017; Pawlowska *et al*., 2018; Okrasińska *et al*., 2021).

In the course of the culture‐based experiment, we isolated several Mucoromycota representatives which were not previously proved to be indicator taxa for any of the delimited groups (compare Figs 4 and 7). Some of them, like *Umbelopsis* sp. 65 (Fig. 7), were present in control, while absent in metal‐ and multi‐polluted soils, and not able to grow on media with BTEX as a sole carbon source, which seems to confirm the hypothesis that some Mucoromycota taxa (e.g. *Umbelopsis*) can be treated as typical for soils that are not anthropogenically transformed (Marfenina, 1999). Other Mucoromycota representatives, like *Mucor* spp. 61–63 and *Umbelopsis* sp. 66 (Fig. 7), were able to grow in the presence of BTEX, but not when it was the only carbon source. Finally, there were also taxa like *Absidia* sp. 13 or 69, which developed on plates with BTEX as a sole carbon source. Interestingly, *Absidia* belongs to the family *Cunninghamellaceae*, same as the genus *Cunninghamella*, which was shown to oxidize PAHs by intracellular cytochrome P450 monooxygenases activity. Our study thus suggests that non‐pathogenic Mucoromycota representatives isolated from hydrocarbon polluted areas, which are known to often interact with bacteria, seem to be the perfect group for further studies of their bioremediation potential.

## Experimental procedures

### Sampling sites

Soil samples were collected in 25 locations in Poland and three in Iran (in total 28 sites) between January 15th and October 30th, 2018 (additional information on the sites and samples can be found in Supplementary Table 1). Two separate 500 g samples of topsoil (A horizon) from each location were collected. In total, 56 samples from 28 sites were collected; however, only 52 of them were included in analysis due to low quality of DNA isolates for four samples (sites represented by single probe are marked by an asterisk in Supplementary Table 1). The fifty‐two analysed samples include 26 from control sites with and without vegetation (18 and 8 probes respectively), eight from metal‐polluted areas (heaps and wastelands around the copper mine) and 18 from multi‐polluted post‐industrial sites (heaps and workings around active and former oil mines or processing plants). Samples from each of the four groups represent similar microhabitat conditions.

### Chemical analysis of soil samples

Fresh soil sample of 200 g from each of 28 locations was collected and sent directly to the laboratory for chemical analysis. The concentrations of selected elements (including heavy metals), i.e. As, Al, Cd, Co, Cr, Cu, Fe, Mo, Mn, Ni, Pb, V and Zn in soil from all sites were determined by inductively coupled plasma optical emission spectrometry. Additionally, concentration of mercury (Hg) was measured using atomic absorption spectroscopy with mercury analyser AMA 254. Metal pollution index (Mi) was calculated according to Lemmel *et al*. (2019). The concentrations of total petroleum hydrocarbons (C6–C12), mineral oils (C12–C35), BTEX [(ethyl)benzene, toluene, three xylene isomers, styrene] and PAHs were also determined using gas chromatography with a mass spectrometer (GC–MS) (for qualitative analysis) and gas chromatography with flame ionization detector (GC‐FID) (for determining the total amounts of petrol, mineral oils and PAHs). All these analyses were performed by Wessling Company (https://pl.wessling-group.com). The carbon, hydrogen, nitrogen and sulfur elemental contents were quantified using a CHNS Elemental Analyser EA1112 (Thermo Finnigan, Italy), and carbon to nitrogen ratio was used as a predictor of site fertility. The presence of vegetation was assessed and encoded as binary variable. The results of chemical analysis are shown in Supplementary Table 1.

### DNA extraction, amplification and sequencing

Total genomic DNA was extracted in five independent biological replicates (each from 0.25 g of homogenized soil) for every sample, using the FastDNA™ Spin Kit for Faeces (MPBio, Carlsbad, CA, USA) according to the manufacturer's protocol. All five DNA isolates from each site were mixed and used as a matrix for the PCR reaction. Each PCR reaction was conducted in triplicate, and subsequently three amplicons were mixed (to minimize the PCR bias) and used for DNA sequencing.

For the preparation of the ITS2 region DNA amplicons, the following primer pair with MiSeq adapters was used: I_ITS3: 5′ TCGTCGGCAGCGTCAGATGTGTATAAGAGACAGGCATCGATGAAGAACGCAGC 3′ and I_ITS4: 5′ GTCTCGTGGGCTCGGAGATGTGTATAAGAGACAGTCCTCCGCTTATTGATATGC 3′ (Tedersoo *et al*., 2014) targeting the ITS2 region. Each reaction was prepared using KAPA HiFi polymerase with appropriate ingredients (KAPA Biosystems) in a T100™ Thermal Cycler (BioRad, CA, USA). After 3 min of initial denaturation of DNA at 95°C, 29 cycles including denaturation (95°C, 30 s.), primer annealing (60°C, 30 s.) and DNA synthesis (72°C, 30 s.) were performed. The last cycle was followed by 5 min of the final DNA extension (72°C).

The 52 amplicon libraries were sequenced using an Illumina MiSeq instrument (Illumina, CA, USA) in the Biobank Lab of the Department of Molecular Biophysics, University of Lodz (Poland), with the use of a v3 MiSeq chemistry kit in the paired‐end mode. Raw sequencing data were deposited in the National Center for Biotechnology Information's Sequence Reads Archive under the project number PRJNA767765.

### Bioinformatic processing of the sequencing data

For fungal diversity analysis, raw Illumina MiSeq paired reads obtained for ITS2 amplicon were processed, using tools and pipelines wrapped by QIIME2 (version 2021.4, Bolyen *et al*., 2019). The DADA2 QIIME2 plugin (Callahan *et al*., 2016) was used to create the ASVs table. Then, the taxonomy of each ASV was assigned using the BLASTn algorithm with default QIIME2 options (Altschul *et al*., 1990) with the UNITE fungal dynamic database with singletons (developer version 8.3) as a reference (Nilsson *et al*., 2019). All further data manipulation and statistics were conducted in RStudio 1.2 (RStudio Team, 2020) with R 3.6.1 (R Core Team, 2020). ASVs obtained with QIIME2 were processed with the LULU R package (Frøslev *et al*., 2017) to remove erroneous ASVs based on all against all BLASTn searches and default LULU settings.

### Diversity analysis

The grouping of soil samples based on logarithmically transformed numeric part of edaphic data was justified by ANOVA test with 999 permutations (on Euclidean distances) and represented using principal component analysis (PCA). Alpha diversity analysis was performed for each sample using Chao1 and Shannon species richness indexes, and Pielou and Simpson evenness measures. The Kruskal–Wallis *H* test was used to estimate the significance of differences between delimited groups.

The rarefied ASVs composition was compared between all sites and represented using NMDS. The significance of differences between delimited groups was justified by ANOVA test with 999 permutations. The correlation between soil parameters and fungal species composition was measured using the Mantel test based on the Euclidean distance matrix.

All above‐mentioned alpha and beta diversity analyses were performed with the application of vegan v2.5.6 (Oksanen *et al*., 2019), microbiome v1.6.0 (Lahti and Sudarshan, 2017), phyloseq v1.28.0 (McMurdie and Holmes, 2013) and reshape v1.4.3 (Wickham, 2007) R packages. Plots were generated with the ggplot2 v3.2.1 (Wickham, 2016) and lemon v0.4.5 (Edwards, 2020) R packages. The .Rmd file is available as Supplementary Material 1.

### Indicator species analysis

The fungal ASVs characteristic for each group was selected using multipatt function (IndVal.g) from indicspecies R package (Caceres and Legendre, 2009). The abundance of selected indicator taxa was shown on heatmap only if (i) the level of statistical significance derived from multipatt function was lower than 0.01 and the ASV was identified at least to the genus level, or (ii) if the ASV remained unidentified but was present in more than one sample and constituted more than 10% of reads in at least one of them. ASVs were considered generalists if they were present in more than half of the samples and were represented by at least five reads in each of them. FungalTraits (Põlme *et al*., 2020) and FUNGuild (Nguyen *et al*., 2016) were used for determining the trophic mode of each genus. The heatmap with the genus level‐identified generalists and specialists was prepared using the phyloseq R package (McMurdie and Holmes, 2013) and InkScape software (Harrington, 2020). Taxonomic assignment and trophic mode of selected indicator species are also shown on heatmap. As several taxa were missing in both FUNGuild and FungalTraits databases, trophic modes were manually assigned based on literature search.

### Selection experiment

The selected soil samples from delimited groups were mixed together as follows: (i) six soil samples from control sites with and without plants (namely, K_BEL_1, P_WJB_1, P_WRJ_1, K_CSL_1, K_ZER_1, P_BEL_1), (ii) three soil samples from metal‐polluted sites (namely, P_LEG_1, P_LEG_2, P_LEG_3) and (iii) three soil samples from multi‐polluted sites (namely, P_BOB_1, P_Ira_13, P_Rop_1). One gram of soil from each site was used for mix preparation. The Warcup soil plate isolation method (Warcup, 1950) was applied using zeolite instead of sand in order to retain volatile compounds during the experiment (i.e. 1.0 g of soil was mixed with 74.0 g of zeolite and plated afterwards). Each of the three above‐mentioned variants of soil mixtures was plated on: (i) 4% malt extract agar (MEA); (ii) 4% MEA supplemented with 1200 ppm of Cu(NO_3_)_2_·H_2_O; (iii) 4% MEA supplemented with 1200 ppm of Cu(NO_3_)_2_·H_2_O, 0.12 ppm of benzene, 0.52 ppm of ethylbenzene, 0.17 ppm of toluene and 9.83 ppm of xylenes mix; and (iv) water agar (WA) with the same addition as in (iii). As BTEX are volatile, they were added to zeolite prior to the Warcup isolation procedure described above. The experiment was performed in five replicates of each variant. For each plate, 0.1 g of the mixture was used. The plates were further incubated for 2 weeks at 17°C. Afterwards, total genomic DNA was extracted jointly from all mycelia overgrowing each plate using the same protocol as in the general experiment (3 soil types × 4 media types × 5 replicates; 60 isolations in total). Each DNA extract was used as a template for three independent PCR reactions. ITS2 amplicons from each soil variant were then mixed and used for library preparation and sequencing. The amplicons were sequenced and analysed as described for the environmental samples. This part resulted in 11 amplicons, as there was no visible growth or PCR product for BTEX‐polluted soil plated on water agar with BTEX and copper salts added.

## Conclusions

The results presented here demonstrate that increasing soil contamination leads to a decrease in overall soil fungal diversity. However, it also enables selection of soil fungal communities adapted to particular types of pollution out of the indigenous inoculum present on‐site. As these organisms possess specific mechanisms enabling them to use particular pollutants as sole energy sources, they should be further studied in order to understand specific molecular mechanisms underlying their adaptation capacities.

In this study, closely related organisms belonging to the same genus have been shown to differ in their ability to use pollutants as the sole carbon source (e.g. *Absidia*). The factors influencing this diversity seem to be crucial in determining the further biotechnological potential of these organisms.

Many fungi are able to partially degrade hydrocarbons when on their own. It is however possible that a cooperative community of various soil microorganisms may completely degrade these pollutants. Therefore, studies on whole communities' bioremediation abilities are needed, rather than single organisms. Still, some of the strains isolated in this study turned out to be able to use particular contaminants as sole energy sources which make them potentially valuable for biotechnological purposes. Further genomic, transcriptomic and proteomic experiments on these strains can lead to describing degradation pathways which can be of use in bioremediation.

## Supporting information


**Supplementary Table 1.** Description: General (physical) and edaphic characteristics of the sites from which samples were taken.

**Supplementary Table 2.** Description: Number of the raw reads before and after each filtration step obtained for each sample for both environmental and culture‐based approach part. The summary numbers for both parts of the study are given in the last rows.

**Supplementary Table 3.** Description: Numbers (absolute and percentages) of the reads assigned to each phylum for each sample from the environmental part of the study.

**Supplementary Material 1.** Description: Rmd file with all the R analyses performed.

**Supplementary Fig. 1.** World contour map with Poland and Iran marked with red dots. In the bottom left there is contour map of Poland, and in the bottom right there is a contour map of Iran, both with the sampling sites marked with red dots.

**Supplementary Fig. 2.** Rarefaction curves for each sample. Vertical line shows the size of the sample with the lowest number of ASVs.
