# Supplementary figures and images for: Marginal lands and fungi – linking the type of soil contamination with fungal community composition
Source: Environ Microbiol. 2022 May 30;24(8):3809–25. doi: 10.1111/1462-2920.16007 (PMC9544152; doi:10.1111/1462-2920.16007)

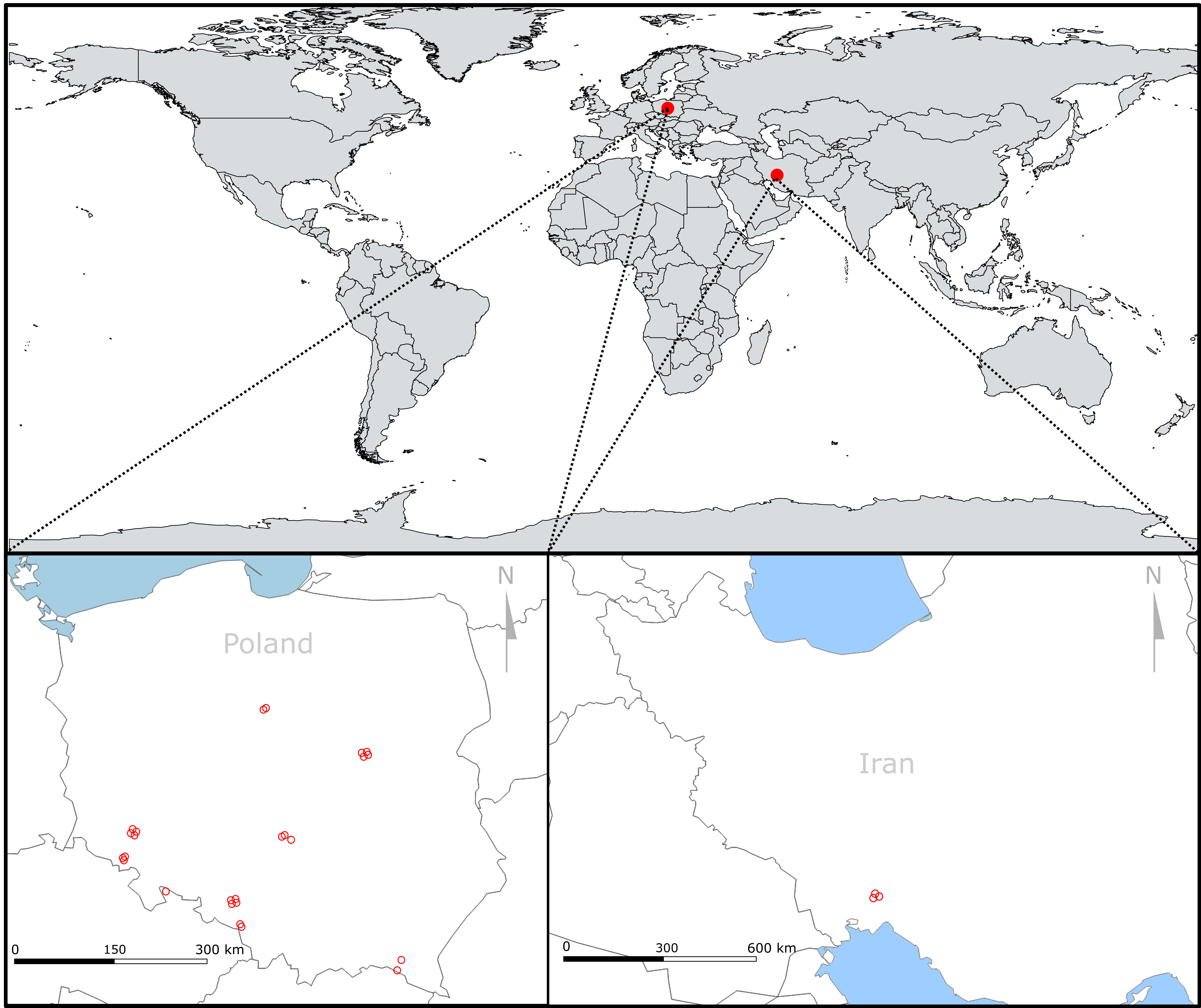

Supplement: Supplementary file 5 — Supplementary Fig. 1. World contour map with Poland and Iran marked with red dots. In the bottom left there is contour map of Poland, and in the bottom right there is a contour map of Iran, both with the sampling sites marked with red dots. [file EMI-24-3809-s003.png]

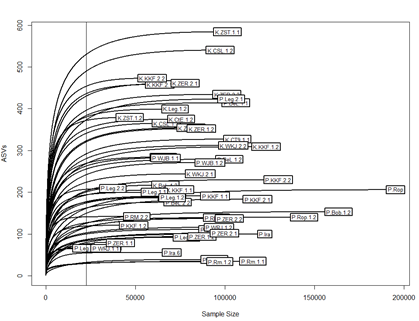

Supplement: Supplementary file 6 — Supplementary Fig. 2. Rarefaction curves for each sample. Vertical line shows the size of the sample with the lowest number of ASVs. [file EMI-24-3809-s001.png]
